# The Canadian dental care plan and the senior population

**DOI:** 10.3389/froh.2024.1385482

**Published:** 2024-06-12

**Authors:** Anil Menon, Robert J. Schroth, Khalida Hai-Santiago, Katherine Yerex, Mary Bertone

**Affiliations:** ^1^ Dr. Gerald Niznick College of Dentistry, Rady Faculty of Health Sciences, University of Manitoba, Winnipeg, MB, Canada; ^2^ Childrens Hospital Research Institute of Manitoba, Winnipeg, MB, Canada; ^3^ Shared Health Manitoba, Winnipeg, MB, Canada; ^4^ School of Dental Hygiene, Dr. Gerald Niznick College of Dentistry, University of Manitoba, Winnipeg, MB, Canada

**Keywords:** seniors, oral health, dental plan, CDCP, Canada, access

## Introduction

In Canada, healthcare (Medicare) is free for citizens, while the private sector predominantly provides and finances dental care. Approximately 60% of individuals receive dental care through employer-provided insurance, while 35% pay for their own care. Public financing only covers dental care for a small percentage of the population, approximately 4%–6%, provided by third-party financing programs. Low-income children and those receiving social assistance may be eligible for these programs. Private and financed dental care arrangements are the norm in Canada, with publicly owned and funded options being uncommon.

## The Canadian dental care plan (CDCP)

In 2019, the United Nations General Assembly reaffirmed its commitment to strengthening and scaling up efforts to address oral health as part of Universal health coverage (UHC) (1). As part of a broader commitment, the Canadian Dental Care Plan (CDCP) is a new federal government program that aims to provide oral health care to eligible Canadian residents. This initiative marks a historic step toward integrating dental care into the broader spectrum of health services, acknowledging oral health's critical role in overall well-being.

This program is significant for seniors in Canada who have historically received inadequate dental care due to factors such as being uninsured or underinsured (2), as well as challenges related to accessing care when mobility and independence decline (3). Seniors may struggle to afford dental care due to rising costs of prescription medications, home heating, food, and gas, along with poor returns on their savings.

Canadian and global research has demonstrated that cost barriers can lead to not obtaining their prescriptions, misusing medications and poor health outcomes (4, 5). Nearly one million Canadians, including seniors, have had to cut back on basic necessities to afford their prescriptions (6). This highlights the challenges seniors on fixed incomes face when balancing essential needs with healthcare expenses. With recent data showing that about 7.5 million seniors (65+) now represent a more significant share of Canada's population than children (7, 8), this plan is timely.

## CDCP eligibility and coverage

The CDCP, a CAD $13-billion program, aims to cover basic dentistry costs for uninsured Canadians, including seniors, with a household income under a certain threshold (Table 1) (9), making it a vital resource for them to obtain necessary dental care without enduring pain or incurring high out-of-pocket expenses. Seniors were the first to be prioritized, with other age groups to follow (9, 10, 11).

**Table 1:** Application timeline for eligible seniors to apply for the CDCP.

Adjusted family net income	CDCP coverage	Senior's coverage
Lower than $70,000	100%	0%
Between $70,000–$79,999	60%	40%
Between $80,000–$89,999	40%	60%

Despite a household income of CAD $90,000 or less, dental insurance coverage disparities and access to healthcare remain major issues for working-poor Canadians, emphasizing the need for CDCP to address these gaps.

## CDCP challenges and opportunities

Several challenges hinder the effective implementation of the CDCP, despite its progressive aims. These include public awareness, the specific wording of the program's title, and the nuanced needs of diverse senior populations.

The effectiveness of the CDCP program depends on how well the general public is aware and informed about its benefits and details. Many dental offices are not fully informed, even after recent updates and information from Health Canada on the application process (1, 9). The medical system also needs to recognize the significance of regular oral health care. It is necessary to inform healthcare providers, associations, health authorities, and community organizations of the program's new plan to address this issue. This will enable them to increase efforts to educate seniors and caregivers about the program and enroll them.

A notable limitation of the program is its name, which utilizes the term “dental” instead of “oral”. This choice of wording may lead to misinterpretation, particularly among edentulous seniors. These individuals might not associate their denture requirements with the broader scope of “dental” care services. Empirical observations have indicated that a segment of the senior population who relies on dentures perceive that they do not necessitate any form of dental care, including the CDCP, attributing this belief to their use of dentures. It's important to educate seniors that regular exams are necessary to observe changes in oral tissues and denture fit.

Cultural barriers, contextual factors and life circumstances, oral health literacy and recent immigration status, particularly for senior immigrants who are parents of recent immigrants, can pose additional challenges in enrollment, and accessing information and services provided by the CDCP. These individuals often face language barriers, have a limited understanding of the Canadian healthcare system, and may not be aware of the available oral health programs (7, 12, 13). Studies have shown that immigrants report poorer oral health outcomes and less utilization of oral health services due to such barriers (2). Incorporating targeted outreach and education efforts, multilingual resources, and culturally sensitive approaches would ensure that these groups are adequately informed and can access the plan's benefits.

While having dental insurance can significantly reduce financial barriers to dental care for seniors, it is important to recognize that these barriers may not be wholly addressed due to varying levels of coverage in the CDCP. Research indicates that universal dental insurance coverage would increase dental care utilization (14), yet the extent would depend on the specific benefits and coverage (14). Also, potentially, employers may discontinue their existing dental benefits in response to implementing the CDCP. Some seniors are working beyond 60, with about 2.7 million people aged 60 and over (representing almost one-third of the population aged 60 and above) who reported working or wanting to work in the previous 12 months (15). Of this, 49% worked or wanted to work out of necessity (15). This scenario could significantly increase the number of individuals depending on the government plan, potentially escalating the program's costs and creating challenges in its management and sustainability.

## Role of dental professionals

A crucial factor in the success of the proposed CDCP is the willingness of dental professionals (dentists, independent practice dental hygienists, and denturists) to accept senior clients under the new remuneration scale. The success of the CDCP depends significantly on their acceptance and support. Therefore, the government needs to engage with dental associations and oral health professionals to ensure that the compensation model is equitable and incentivizes participation by the oral health community. Presently, there appears to be resistance from dentists and their associations in Canada (16, 17). Since participation in the CDCP is at providers' discretion, it raises critical questions regarding the alternatives available to patients should their regular dental care providers choose not to participate. This situation may compel patients to find new providers, potentially impacting the fairness and accessibility of the plan. Another concern is that the compensation across provinces and territories based on the dental services fee guides varies, which could lead to discrepancies in care and coverage, affecting both patients and providers.

Providers and offices should also receive guidance on how the CDCP will harmonize with existing public insurance programs like the Non-Insured Health Benefits program and provincial Employment and Income Assistance. This harmonization is crucial for provider enrollment, as it affects their practice's operational and financial aspects and determines how they manage patients covered under different insurance schemes.

## Impact on senior oral health and reaching beyond ambulatory care

The CDCP needs to emphasize the importance of preventive care and tailored recall intervals. This approach should be based on individual risk assessments, potentially leading to more frequent dental or hygiene visits for those at higher risk of severe dental conditions. Incorporating measures like fluoride varnish and silver diamine fluoride (SDF) can significantly contribute to maintaining oral health in the senior population, thereby reducing the incidence and severity of dental issues (18). Tailoring recall intervals based on risk assessments align with current best practices in dental public health and could improve the overall effectiveness of the CDCP.

The current CDCP structure primarily caters to mobile seniors who can visit dental clinics. This approach overlooks the segment of seniors residing in personal care homes who may have limited or no mobility, varying levels of dementia and require on-site oral health care (19), including varying levels of comorbidities. Providing dental care in personal care homes entails logistical and financial complexities, including setting up mobile operatory units, adapting to confined spaces, and allocating additional time for each visit (19).

Also, onsite care in personal care homes involves additional regulations (20), which significantly increase the effort and cost required to deliver care. The pay structure may not account for the extra time and resources needed, leading to a lack of incentives for dental professionals. This oversight could lead to a lack of incentive for dental professionals to offer these essential services to non-mobile seniors in their own homes or personal care homes, further delaying treatment in high-needs populations.

## Discussion

To ensure equitable access to dental care for all seniors, including those in personal care homes, the CDCP needs to expand its coverage thoughtfully. As the Federal government finalizes its decision-making around what services will be covered as part of the CDCP, it must consider including specific provisions for on-site dental care in personal care homes and survey the provider group to get feedback on special considerations for caring for this population. This revision should include an enhanced pay structure for dental professionals providing these services, ensuring they are adequately compensated for their additional challenges by either having a different fee guide with higher reimbursement rates or allowing dental providers to bill a house call fee per visit (Table 2). Another suggestion would be to integrate mid-level dental providers, such as advanced dental hygiene practitioners, to perform basic restorative and preventive care and help extend services to underserved areas.

**Table 2:** Considerations for enhancing oral health services for seniors through the CDCP.

Clinical oral health services	Supplemental preventive products	Incentives for oral health professions	Grants and subsidies	Additional considerations
• Fluoride varnish (covered, but need to see if the frequency of applications is not restricted) • Silver Diamine Fluoride (SDF) (covered, but need to see if the frequency of applications is not restricted) • Frequent recalls • Frequent hygiene care and maintenance (up to 4 units covered without predetermination and more with predetermination)	• 5,000 ppm fluoridated toothpaste or gel • Electric toothbrushes or adaptive devices for those with dexterity issues	• Increased fees for care in personal care homes • House call fee code for care in personal care homes (covered, but predetermination is required)	• Grants for additional services • Subsidies for preventive products • Incentives for remote area care • Funding for educational programs • Training providers to treat the senior population	• Annual oral cancer screenings • Radiographs as needed based on risk assessment • Specialized treatments for dry mouth conditions • Prescription-strength mouthwashes for gum disease • Interdental brushes or floss for improved interdental cleaning • Denture care and cleaning products for those with removable prosthetics • Subsidies for continuing education focused on geriatric dentistry • Grants for research in geriatric oral health care • Denture labelling to aid identification

Additionally, including more comprehensive coverage for fluoride varnishes and silver diamine fluoride (SDF) (Table 2) (18) and assistance with the cost of high fluoride concentration toothpaste is vital for seniors, especially those in personal care homes. These individuals often face increased risks of dental caries and other oral health issues due to age-related factors like diminished saliva production (due to multiple medications) and manual dexterity challenges, impacting proper mouth care (21, 22). The American Dental Association's clinical guidelines advocate for the non-restorative management of carious lesions, emphasizing the effectiveness of preventive therapies like SDF and fluoride varnishes (23). These treatments are crucial in managing and arresting dental decay, reducing the need for more invasive and costly dental procedures, for seniors with limited access to dental care and reliance on others to provide daily mouth care. A fee structure should also be implemented for non-dental providers who use oral health screening tools to screen vulnerable populations. This aligns with best practices for maintaining oral health and should be a crucial component of the CDCP.

Beyond the CDCP alone, the oral health community and educational institutions will need to ensure that there will be enough current and future providers across the country to increase this group's access to oral health care. The CDCP is bolstered by the introduction of the Oral Health Access Fund, as detailed in the 2023 Budget, which dedicates a substantial investment of $250 million over a three-year period commencing in 2025–26, followed by an ongoing allocation of $75 million per annum (24). The fund aims to address oral health disparities in underserved regions, including remote and rural areas, by improving the accessibility of dental care. Community-based organizations and educational institutions can contribute through various approaches, such as creating educational programs and scholarships for dental professionals, building community outreach and awareness programs, equipping non-dental health workers with basic oral healthcare and preventive strategies, and utilizing teledentistry initiatives.

The CDCP stands on the verge of change, promising a future where dental care is not a privilege but a fundamental right, regardless of age or socioeconomic status. While the CDCP is a significant step forward, addressing these gaps is crucial to making the program truly inclusive and effective in improving the oral health of all seniors in Canada. All stakeholders must work together to ensure that the program serves its intended purpose of providing equitable access to oral health care for all eligible residents. It is also crucial that the public and seniors are given timely and accurate information about CDCP, what dental services are covered and potential fee structures. The insights gained from implementing the CDCP will be invaluable for other nations considering the adoption of universal oral health care for their citizens.
